# Ecological relationships of *Haemaphysalis longicornis* Neumann with other tick species on wildlife hosts at cow–calf farms implementing integrated pest management in eastern Tennessee

**DOI:** 10.1017/S0031182024001380

**Published:** 2024-08

**Authors:** Rebecca A. Butler, Lisa I. Muller, Dan Grove, Rebecca T. Trout Fryxell

**Affiliations:** 1 Department of Entomology and Plant Pathology, University of Tennessee, Knoxville, TN, USA; 2 School of Natural Resources, University of Tennessee, Knoxville, TN, USA

**Keywords:** exotic, *Haemaphysalis longicornis*, integrated pest management, invasive, wildlife–livestock interface

## Abstract

Longhorned tick (*Haemaphysalis longicornis*) is an invasive tick species that parasitizes a variety of vertebrate hosts and transmits pathogens affecting humans and livestock in the United States (US). Unfortunately, the behaviour of this tick at the wildlife–livestock interface is not well understood. To better understand how *H. longicornis* uses wildlife hosts and interacts with established tick species on farm settings we sampled small and medium wildlife seasonally for a year, using Sherman and Tomahawk traps, on three *H. longicornis*-infested cattle farms in eastern Tennessee. We confirmed that wildlife host body regions and coinfesting tick species were associated with the likelihood that *H. longicornis* would be present on a host. In addition, ticks were less likely to be present on hosts when farmer led integrated pest management strategies were adopted and the environment was modified to reduce tick populations. Results from this study can be used to target host species for on-animal management of *H. longicornis* by using population management strategies or acaricidal applications. Activity patterns for when established tick species, with similar predicted geographic ranges as *H. longicornis*, are feeding simultaneously on hosts can also be used to predict when this exotic tick species will be present. Finally, reducing tick abundance in the environment can be important for on-animal control. These results are imperative for understanding how wildlife hosts harbour *H. longicornis* and its interactions with established tick species. These findings are useful for selecting tick management strategies specific to *H. longicornis* and understanding pathogen transmission due to cofeeding.

## Graphical abstract

**Figure:**
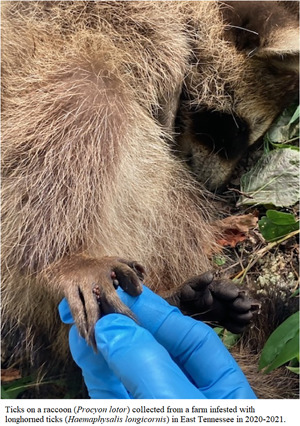

## Introduction

The introduction of exotic and invasive vectors into the United States (US) is not only a concern for their negative ecological and economic effects (Marbuah *et al*., 2014; Bradshaw *et al*., 2016), but also for potential health impacts (Lounibos, 2002; Juliano and Philip Lounibos, 2005). Several tick species of great concern, due to their potential negative impact on the livestock industry, have invaded the US, including the red sheep tick *Haemaphysalis punctata* (Tufts and Diuk-Wasser, 2021), cattle fever ticks, *Rhipicephalus* (*Boophilus*) *annulatus* (Say) and *Rhipicephalus* (*Boophilus*) *microplus* (Canestrini) (Lohmeyer *et al*., 2011), and the longhorned tick *H. longicornis* (Beard *et al*., 2018). The recent discovery of *H. longicornis* in the US is a public health concern due to its ability to transmit a variety of pathogens that impact humans and other animal hosts (Thompson *et al*., 2021). Although *H. longicornis* is not known to use humans as a preferred host (Ronai *et al*., 2020), it is able to harbour and transmit pathogens which negatively affects humans (Zhuang *et al*., 2018; Stanley *et al*., 2020). Reports in the US found *H. longicornis* parasitizing a wide array of mammal and bird species (USDA-APHIS, 2023). Previous studies surveying *H. longicornis* on wildlife hosts found white-tailed deer (*Odocoileus virginiana*), raccoon (*Procyon lotor*) and Virginia opossum (*Didelphis virginiana*) to be heavily infested with this tick species (Thompson *et al*., 2021, 2022). Although this tick has been widely reported on companion and livestock animals (USDA-APHIS, 2023), its role in the transmission of pathogens from wildlife to humans and domesticated animals in the US is likely underreported. Cofeeding, attachment of 2 separate tick species feeding together on a host, between *H. longicornis* and other native tick species on companion and wildlife hosts in the US have been reported (Tufts *et al*., 2019, 2021). Cofeeding ticks can have negative implications for human health because they can obtain pathogens from one another; thus, *H. longicornis* may obtain pathogens from native tick species or vice versa (Tufts, 2021; Price *et al*., 2022). For example, Bourbon virus and *Anaplasma phagocytophilum* from native cofeeding ticks were found in *H. longicornis*, which could have been transmitted by ticks feeding in proximity (Cumbie *et al*., 2022; Price *et al*., 2022). Trout Fryxell *et al*. (2023) found *H. longicornis* follows similar phenological patterns to native tick species and these species were often found questing together in similar habitats. It is possible that native species could be interacting with *H. longicornis* in the environment as well as on hosts. Knowing how exotic and native tick species select host species is important for understanding pathogen transmission.

To better understand the community structure of *H. longicornis* on hosts at wildlife–livestock interfaces this study aimed to identify this species' associations with established tick species and wildlife hosts and producer led integrated pest management (IPM) strategies in eastern Tennessee. Although the goal of the present study was to identify factors associated with *H. longicornis* abundance and presence, which could be targeted for wildlife on-animal control on farms infested with this tick species, important findings for other tick species were also included. Here, it was hypothesized that established tick species and wildlife host characteristics (e.g. sex, age and weight) and body regions would be associated with *H. longicornis* abundance and presence, and producer led IPM would decrease the presence of this species on wildlife hosts. Factors identified from this research will be important for cost-effective management regimes for controlling *H. longicornis* on cow–calf farm settings.

## Materials and methods

### Site selection

Mammals were trapped in forested and edge habitats on 3 farms (farm 1, farm 2, farm 3), which consisted of upland hardwoods surrounded by fields of fescue in eastern Tennessee. Each farm had an established population of *H. longicornis* on site and a herd of beef cattle. Producer led IPM strategies were conducted on site at each farm and outlined in Butler and Trout Fryxell (2023). These on-farm management strategies consisted of chemical control in the form of an on-animal acaracide for cattle, cultural control in the form of bush hogging pastures, and mechanical control by removing ticks, in forested, field, and edge habitats, from the environment using a corduroy drag. Farm 1 significantly reduced questing *H. longicornis* in the environment by 90% over 3 years; they bush hogged their pastures once monthly during the growing season (May–October), used an on-animal acaracide, and frequently removed ticks from the environment by mechanical control. Farm 2 did not reduce questing *H. longicornis*; they chose to bush hog their pasture once a year in the fall, did not use an on-animal acaracide, and moderately removed ticks from the environment by mechanical control. Farm 3 reduced their *H. longicornis* population by 68%; that producer bush hogged their pastures yearly in the fall, used an on-animal acaracide, and scarcely removed ticks from the environment by using mechanical control (Butler and Trout Fryxell, 2023).

### Mammal handling and tick collection

All wildlife were handled following guidelines of the American Society of Mammalogists (Sikes and Gannon, 2011) and identified to species using taxonomic keys outlined in Kellogg (1939); Schwartz and Schwartz (2016). Methods for handling small- and medium-sized wildlife were approved by the University of Tennessee, Knoxville (IACUC #2774-0620). Species, sex, age (subadult and adult), weight (g) and external morphological measurements (mm) were taken on each wildlife host. Small mammals were trapped using Sherman live traps (2″ × 2.5″ × 6.5″, H. B. Sherman Traps, Inc., Tallahassee, FL, USA) baited with rolled oats and black oil sunflower seeds (Owen *et al*., 2015; Butler *et al*., 2020). Medium-size mammals were trapped using medium (16.5″ × 5.75″ × 5.75″, Tomahawk Live Trap, Hazelhurst, WI, USA) and large (32″ × 10″ × 12″, Tomahawk Live Trap) tomahawk traps baited with sardines and marshmallows (Modarelli *et al*., 2020). In 2020–2021, a total of 42 Sherman live traps, 14 medium-sized tomahawk, and 10 large tomahawk traps were placed in forested and edge habitats on each farm. Traps were run for 2-week intervals in every season, which was determined by equinox and solstice, to accommodate differences in tick–host feeding patterns determined by day length (Trout Fryxell *et al*., 2023). Mammals were trapped in every season to understand *H. longicornis* on-host phenology throughout the year. Raccoons were sedated with 20 mg kg^−1^ ketamine and 4 mg kg^−1^ xylazine administered by intramuscular (IM) injection and reversed with 0.375 mg kg^−1^ atipamezole IM (Eiden *et al*., 2015; Johnson *et al*., 2024). Opossums were sedated with 10 mg kg^−1^ ketamine and 2 mg kg^−1^ xylazine IM, but a reversal was not used (Stoskopf *et al*., 1999; Kreeger and Arnemo, 2018). Small mammals (rodents and squirrels) were sedated in an enclosed plastic box with cotton balls of isoflurane and monitored for sedation every 5 s or until the animal became drowsy (Bennett and Lewis, 2022). Ticks were removed from trapped mammal hosts with forceps and stored in 80% ethanol. All ticks were identified to species and life stage using taxonomic keys but were not sized for engorgement status (Yunker *et al.*, 1986; Keirans *et al*., 1996; Keirans and Durden, 1998; Egizi *et al*., 2019).

### Statistical modelling

**Figure 1.**
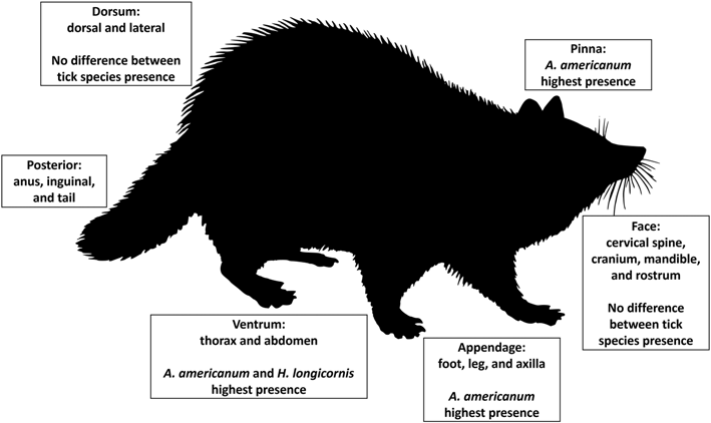
Morphological body regions where ticks were collected on small and medium wildlife hosts on farms in eastern Tennessee in 2020–2021. In addition, significant associations for tick species, *Amblyomma americanum*, *Haemaphysalis longicornis*, *Dermacentor variabilis,* and *Ixodes scapularis*, were labelled for each body region.

All analyses were performed in Statistical Analysis Software (SAS, ver. 9.4, Cary, NC, USA) with 2-tailed hypotheses (*α* = 0.05). The best fit model was chosen based on minimizing AIC and BIC as well as the −2 restricted (res) log likelihood statistics. Least squares means (LS Mean) Tukey–Kramer post-hoc pairwise comparisons were used for examination of each categorical variable. All mammal hosts collected were included in each model; further, season and tick life stage were not blocked on due to low sample sizes associated with some categorical variables. A generalized linear mixed model (PROC GLIMMIX) with negative binomial distribution was used to model associations between response variable tick abundance and the presence of each tick species, host morphological body region where ticks were collected and their interaction as a main effect (tick species × body region). Collected ticks were categorized based on host morphological body regions appendage for ticks collected on the foot, leg, or axilla; ventrum for the thorax and abdomen; face for cervical spine, cranium, mandible and rostrum; posterior for anus, inguinal and tail; dorsum for dorsal and lateral regions; and pinna for the external ear regions (Fig. 1). Posterior region was dropped from all models due to low sample sizes. Models used to analyse tick interactions based on morphological body regions used each body part as the unit of observation. Each individual animal was used as a random effect in the model and the variables management type and mammal type were used as covariates. Mammal type represents small or medium mammals and management refers to the tick management strategy used on each farm following Butler and Trout Fryxell (2023). In addition, a hierarchical linear mixed model (PROC GLIMMIX) with a negative binomial distribution was built using each individual animal as a random effect with mammal type and management as covariates. Shannon index of species diversity was used as the response variable *H* to identify the diversity of tick species across host body regions using the formula (Shannon, 1948; Dejong, 1975), *K* as a constant and *p*_*i*_ is the proportion of the entire body region made up of species *i*. *p*_*i*_ was calculated by dividing the number of ticks collected on each body region by the total sum of ticks collected. Shannon index of species diversity was also calculated for the diversity of tick species on each mammal host.
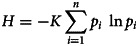

Chi-squared (PROC FREQ) analyses were then run to understand interactions between each tick species occurring on host body region and *P* values were determined. Additionally, odds ratios and their 95% confidence intervals were examined for each tick species. Finally, a generalized linear model (PROC GLIMMIX) with a negative binomial distribution was constructed with each individual mammal as the unit of observation. This was done to understand relationships between *H. longicornis* abundance as a dependent variable and the total tick species present and management strategy used by each farm. Host characteristics sex, age, and weight did not contribute to model performance and were removed.

## Results

**Figure 2.**
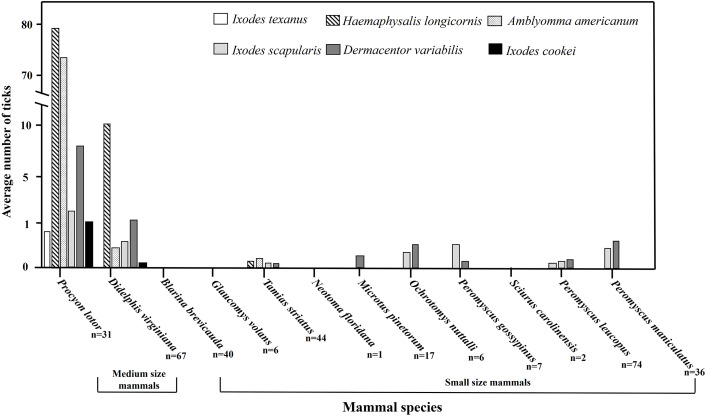
Average number of ticks collected on wildlife hosts on farms in East Tennessee and the total number of hosts collected, denoted as (*n* = ) under scientific name, in 2020–2021.

There was a total of 11 088 trap nights resulting in the collection of 329 wildlife hosts comprised of 11 different mammal species with 6040 ticks on farms in eastern Tennessee. Farm 1 had 800 (13.24%) ticks on 31 mammal hosts out of 89 collected, farm 2 had 184 ticks (3.04%) on 21 mammal hosts out of 100 collected, and farm 3 had 5056 (83.70%) ticks on 52 wildlife hosts out of 140 collected. Overall, 40 northern short-tailed shrews (*Blarina brevicauda*), six southern flying squirrels (*Glaucomys volans*), one eastern woodrat (*Neotoma floridana*) and two eastern grey squirrels (*Sciurus carolinensis*) were captured but zero ticks were present on them (Table 1; Fig. 2). There were 65 Virginia opossums with 843 ticks (mean ± standard error of the mean) (Shannon diversity index) (12.9 ± 4.59) (0.704) and 31 raccoons with 5096 ticks (164.3 ± 53.35) (0.964) collected (Table 1; Fig. 2). Seventeen woodland voles (*Microtus pinetorum*) had a total of four ticks (0.2 ± 0.18) and a diversity index could not be calculated. Six golden mice (*Ochrotomys nuttalli*) with five ticks (0.8 ± 0.83) (0.673), seven cotton deermice (*Peromyscus gossypinus*) with five ticks (0.7 ± 0.28) (0.500), 74 white-footed deermice (*Peromyscus leucopus*) with 21 ticks (0.2 ± 0.07) (0.878), 36 North American deermice (*Peromyscus maniculatus*) with 38 ticks (1.0 ± 0.34) (0.680), and 44 eastern chipmunks (*Tamias striatus*) with 28 ticks (0.6 ± 0.38) (1.272) were collected (Table 1; Fig. 2). There were as few as one and as many as six tick species found feeding together on infested wildlife hosts.

**Table 1.** Descriptive statistics depicting the mean **±** standard error (minimum range–maximum range) and total number of ticks separated by tick species and life stage for wildlife hosts collected on farms in East Tennessee in 2020–2021

	Host species mean ± standard error (range) total number of ticks							
Tick species and life stage	*Procyon lotor* (*n* = 31)	*Didelphis virginiana* (*n* = 67)	*Tamias striatus* (*n* = 44)	*Peromyscus leucopus* (*n* = 74)	*Peromyscus maniculatus* (*n* = 36)	*Microtus pinetorum* (*n* = 17)	*Ochrotomys nuttali* (*n* = 6)	*Peromyscus gossypinus* (*n* = 7)
*Haemaphysalis longicornis*								
Larva	75.8 ± 37.47 (0–871) 2350	9.2 ± 4.18 (0–193) 599	0.0 ± 0.02 (0–1) 1	0	0	0	0	0
Nymph	3.2 ± 0.76 (0–16) 101	0.9 ± 0.27 (0–9) 62	0.1 ± 0.09 (0–4) 5	0	0	0	0	0
Female	0.0 ± 0.04 (0–1) 2	0.1 ± 0.12 (0–8) 11	0	0	0	0	0	0
**Total ticks**	**79.1 ± 37.51** (**0–875) 2,453**	**10.3 ± 4.59** (**0–197) 672**	**0.1 ± 0.10 (0–4) 6**	**0**	**0**	**0**	**0**	**0**
*Amblyomma americanum*								
Larva	64.1 ± 20.52 (0–523) 1,990	0.3 ± 0.22 (0–14) 21	0.1 ± 0.13 (0–6) 6	0.0 ± 0.02 (0–2) 2	0	0	0	0
Nymph	9.6 ± 1.63 (0–37) 300	0.0 ± 0.03 (0–1) 5	0.1 ± 0.15 (0–7) 7	0	0	0	0	0
Male	0.1 ± 0.07 (0–2) 3	0.0 ± 0.01 (0–1) 1	0	0	0	0	0	0
**Total ticks**	**73.9 ± 20.76** (**0–533) 2,293**	**0.4 ± 0.22** (**0–14) 27**	**0.3 ± 0.29 (0–13) 13**	**0.0 ± 0.02 (0–2) 2**	**0**	**0**	**0**	**0**
*Dermacentor variabilis*								
Larva	0	0.0 ± 0.02 (0–1) 2	0.0 ± 0.03 (0–1) 2	0.1 ± 0.05 (0–3) 9	0.5 ± 0.23 (0–5) 21	0.2 ± 0.18 (0–3) 4	0.3 ± 0.33 (0–2) 2	0.1 ± 0.14 (0–1) 1
Nymph	0	0	0	0.0 ± 0.03 (0–2) 4	0.0 ± 0.02 (0–1) 1	0	0.1 ± 0.16 (0–1) 1	0
Male	4.5 ± 1.61 (0–41) 140	0.9 ± 0.30 (0–14) 60	0.0 ± 0.04 (0–2) 2	0	0	0	0	0
Female	2.6 ± 0.97 (0–22) 82	0.6 ± 0.18 (0–6) 41	0	0	0	0	0	0
**Total ticks**	**7.1 ± 2.50** (**0–63) 222**	**1.5 ± 0.46** (**0–19) 103**	**0.0 ± 0.05 (0–2) 4**	**0.1 ± 0.06 (0–2) 13**	**0.6 ± 0.23 (0–5) 22**	**0.2 ± 0.18 (0–3) 4**	**0.5 ± 0.50 (0–3) 3**	**0.1 ± 0.14 (0–1) 1**
*Ixodes scapularis*								
Larva	2.0 ± 0.72 (0–14) 64	0.3 ± 0.17 (0–8) 22	0.0 ± 0.03 (0–1) 2	0.0 ± 0.03 (0–2) 4	0.4 ± 0.28 (0–10) 16	0	0	0.5 ± 0.29 (0–2) 4
Nymph	0.2 ± 0.11 (0–3) 8	0.2 ± 0.07 (0–3) 14	0.0 ± 0.03 (0–1) 3	0.0 ± 0.01 (0–1) 2	0	0	0.3 ± 0.33 (0–2) 2	0
Female	0	0.0 ± 0.02 (0–1) 3	0	0	0	0	0	0
**Total ticks**	**2.3 ± 0.79** (**0–15) 72**	**0.6 ± 0.18** (**0–8) 39**	**0.1 ± 0.04 (0–1) 5**	**0.0 ± 0.03 (0–2) 6**	**0.4 ± 0.28 (0–10) 16**	**0**	**0.3 ± 0.33 (0–2) 2**	**0.5 ± 0.29 (0–2) 4**
*Ixodes cookei*								
Larva	0.4 ± 0.88 (0–3) 13	0.0 ± 0.12 (0–1) 1	0	0	0	0	0	0
Nymph	0.6 ± 1.76 (0–8) 19	0.0 ± 0.12 (0–1) 1	0	0	0	0	0	0
Female	0.0 ± 0.25 (0–1) 2	0	0	0	0	0	0	0
**Total ticks**	**1.1 ± 0.44** (**0–12) 34**	**0.0 ± 0.02** (**0–1) 2**	**0**	**0**	**0**	**0**	**0**	**0**
*Ixodes texanus*								
Larva	0.0 ± 0.35 (0–2) 2	0	0	0	0	0	0	0
Nymph	0.3 ± 1.19 (0–6) 10	0	0	0	0	0	0	0
Female	0.3 ± 1.04 (0–5) 10	0	0	0	0	0	0	0
**Total ticks**	**0.7 ± 1.97 (0–9) 22**	**0**	**0**	**0**	**0**	**0**	**0**	**0**
**Total ticks**	**5096**	**843**	**28**	**21**	**38**	**4**	**5**	**5**

40 *Blarina brevicada*, 6six*Glaucomys volans*, two *Sciurus carolinensis,* and one *Neotoma floridana* were searched, but no ticks were recovered from those animals.

No male *Haemaphysalis longicornis*, *Ixodes scapularis*, *Ixodes cookei* or *Ixodes texanus*, or female *Amblyomma americanum* were collected.

*Haemaphysalis longicornis* were recovered from raccoon more than any other host species; however, Virginia opossum was the second most frequented host and eastern chipmunk was the third (*F*_2, 137_ = 3.75, *P* < 0.0001). *Amblyomma americanum* was most abundant on raccoon and second most abundant on Virginia opossum and eastern chipmunk, while the tick was least abundant on the white-footed deer mouse (*F*_3, 210_ = 38.97, *P* < 0.0001). *Dermacentor variabilis* was most abundant on raccoon; second most abundant on Virginia opossum and North American deer mouse (*F*_7, 272_ = 10.29, *P* < 0.0001); third most abundant were golden mouse, white-footed deer mouse, woodland vole, and cotton deer mouse; and least abundant on eastern chipmunks (*F*_7, 272_ = 10.29, *P* < 0.0001). *Ixodes scapularis* was most abundant on raccoon and Virginia opossum; second most abundant on cotton deer mouse, North American deer mouse, and golden mouse; and least abundant on eastern chipmunk and white-footed deer mouse (*F*_6, 256_ = 5.92, *P* < 0.0001). *Ixodes cookei* was most abundant on raccoon and second on Virginia opossum (*F*_1, 94_ = 17.96, *P* < 0.0001), whereas *I. texanus* was only found on raccoon.

### Tick species cofeeding interactions differed by host body region

**Figure 3.**
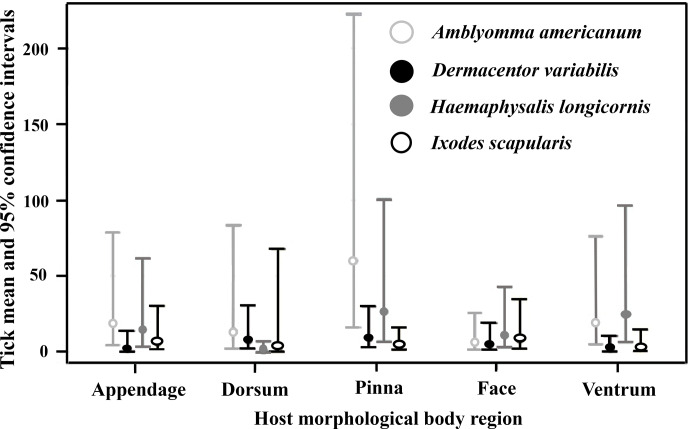
Mean and 95% confidence intervals for where tick species, *Amblyomma americanum*, *Dermacentor variabilis*, *Haemaphysalis longicornis,* and *Ixodes scapularis*, were collected on wildlife hosts, *Procyon lotor*, *Didelphis virginiana*, *Tamis striatus*, *Microtus pinetorum*, *Ochrotomys nuttalli*, *Peromyscus leucopus*, *P. gossypinus,* and *P. maniculatus*, morphological body regions on farms in East Tennessee in 2020–2021.

**Figure 4.**
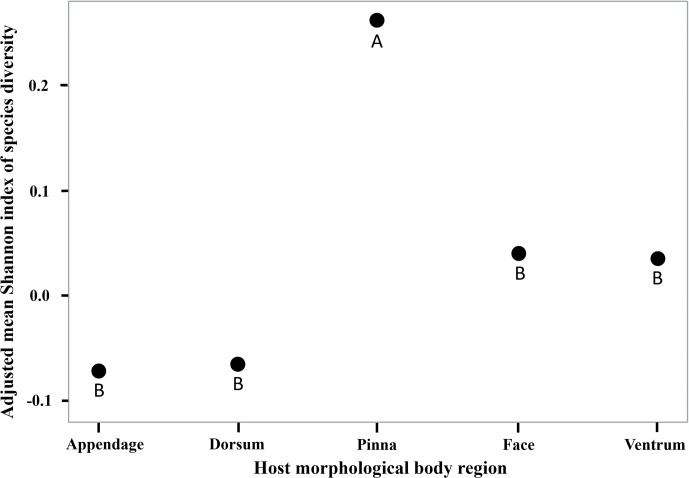
Adjusted mean Shannon diversity index for tick species, *Amblyomma americanum*, *Dermacentor variabilis*, *Haemaphysalis longicornis,* and *Ixodes scapularis*, collected on wildlife hosts, *Procyon lotor*, *Didelphis virginiana*, *Tamis striatus*, *Microtus pinetorum*, *Ochrotomys nuttalli*, *Peromyscus leucopus*, *P. gossypinus,* and *P. maniculatus*, morphological body regions on farms in East Tennessee in 2020–2021. Pinna regions (A) had significantly more diverse tick species compared to host appendage, dorsum, face, or ventrum (B).

Interactions between tick species abundance × host morphological body regions for all host species (*F*_12, 201_ = 3.75, *P* < 0.0001) were associated with tick abundance (Table 2). On appendage and pinna regions *A. americanum* had the highest abundance. There was no difference between *A. americanum*, *D. variabilis*, *H. longicornis,* or *I. scapularis* on the dorsum or face regions. Finally, *A. americanum* and *H. longicornis* had the highest abundance on the ventrum region (Figs 1 and 3). Tick diversity was associated with the host body region where they were feeding (*F*_4, 131_ = 8.90, *P* < 0.0001). Pinna regions had the highest overall adjusted average diversity, but face and ventrum regions had a higher diversity compared to appendage and dorsum regions (Fig. 4).

**Table 2.** Total number of ticks, separated by species and life stage, collected on wildlife host's body regions on farms in East Tennessee in 2020–2021

c						
Tick life stage	Pinna	Face	Appendage	Dorsum	Ventrum	Posterior
*Haemaphysalis longicornis*						
Larvae	1968	78	21	0	524	78
Nymphs	87	24	5	4	27	4
Females	6	1	0	1	5	0
**Total ticks**	**2061**	**103**	**26**	**5**	**556**	**82**
*Amblyomma americanum*						
Larvae	1739	28	32	12	159	32
Nymphs	116	27	113	1	34	4
Males	0	1	1	0	0	0
Females	0	0	0	0	0	0
**Total ticks**	**1855**	**56**	**146**	**13**	**193**	**36**
*Dermacentor variabilis*						
Larvae	38	1	0	1	1	0
Nymphs	1	0	0	3	0	0
Males	156	15	1	15	2	1
Females	77	3	2	32	2	2
**Total ticks**	**272**	**19**	**3**	**51**	**5**	**3**
*Ixodes scapularis*						
Larvae	55	25	19	0	5	0
Nymphs	14	6	2	1	2	0
Males	0	0	0	0	0	0
Females	2	0	0	0	0	0
**Total ticks**	**71**	**31**	**21**	**1**	**7**	**0**
*Ixodes_cookei*						
Larvae	8	3	0	0	2	0
Nymphs	12	8	0	0	0	0
Males	0	0	0	0	0	0
Females	0	1	1	0	0	0
**Total ticks**	**20**	**12**	**1**	**0**	**2**	**0**
*Ixodes texanus*						
Larvae	2	0	0	0	0	0
Nymphs	3	7	0	0	0	0
Males	0	0	0	0	0	0
Females	1	7	0	1	0	0
**Total ticks**	**6**	**14**	**0**	**1**	**0**	**0**
**Total ticks overall**	**4285**	**235**	**197**	**71**	**763**	**121**

*Amblyomma americanum* 17 larvae, 17 nymphs, and 2 males; *Dermacentor variabilis* 2 nymphs, 12 males, and 5 females; *Haemaphysalis longicornis* 281 larvae and 17 nymphs; *Ixodes scapularis* 8 larvae, 4 nymphs and 1 female; *Ixodes cookei* 1 larvae; and *Ixodes texanus* 1 female did not have the host body region recorded.

### Ticks cooccurred differently on wildlife hosts depending on species

**Figure 5.**
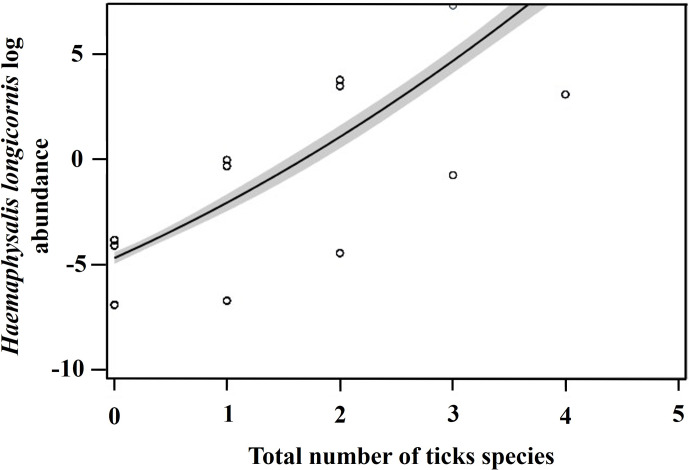
The log abundance of *Haemaphysalis longicornis* on wildlife hosts and the number of total tick species on farms in East Tennessee in 2020–2021.

The likelihood of finding ticks on different regions when other tick species were present was also compared. There was a 37% decrease in the odds of *D. variabilis* being present on regions when and where *A. americanum* was also present (*P* value = 0.0011; odds ratio 0.3728; 95% Cl 0.2048–0.6787) and a 53% decrease in the odds of *H. longicornis* being present on a host's body region when *D. variabilis* was present (*P* value = 0.0315; odds ratio 0.5319; 95% Cl 0.2990–0.9465). However, there was a 209% increase in the odds of *H. longicornis* being present whenever *A. americanum* was also present (*P* value = 0.0002; odds ratio 3.0932; 95% Cl 1.7002–5.6275). Additionally, *H. longicornis* abundance was 45 times more likely to increase with a 1 unit increase in the number of tick species on a host (*F*_1, 325_ = 13.11, *P* < 0.0001) (Fig. 5).

### Pasture management implications reduced ticks on wildlife

**Figure 6.**
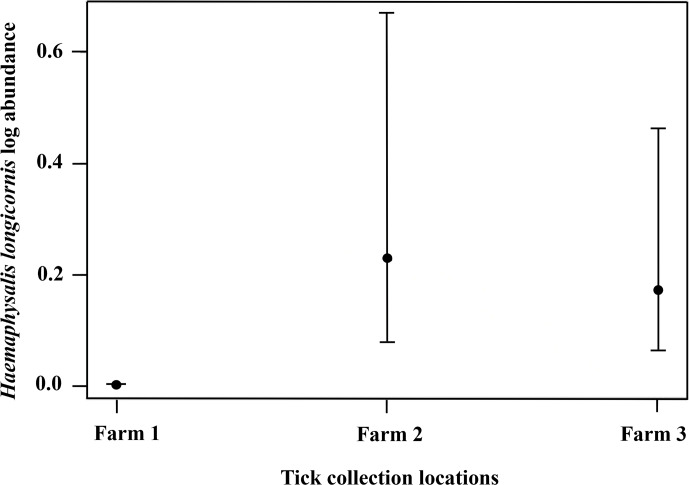
The abundance of *Haemaphysalis longicornis* on wildlife hosts at three farms in East Tennessee in 2020–2021. Each farm adopted different integrated pest management techniques to control tick populations in the environment which ultimately affected the abundance on wildlife hosts. Farm 1 bush hogged monthly, dragged for ticks frequently, and used on-animal control for cattle; farm 2 used bush hogged yearly, dragged for ticks moderately, and used on-animal control for cattle; farm 3 bush hogged yearly, dragged for ticks scarcely, and did not use an on-animal control for cattle.

The management strategy used by each producer was also associated with *H. longicornis* infesting wildlife or on-animal abundance (*F*_2, 325_ = 13.11, *P* < 0.0001), where higher numbers were found on farms 2 and 3 compared to farm 1 but there was no difference in the number of ticks collected from farms 2 and 3 (Fig. 6). On farm 1 there were 7 total (3 larvae and 4 nymphs) *H. longicornis* found on one raccoon and five Virginia opossum. Farm 2 had 93 (68 larvae, 24 nymphs and 1 female) *H. longicornis* on three raccoons and seven Virginia opossum, and farm 3 had 3031 (2879 larvae, 140 nymphs and 12 females) *H. longicornis* on two eastern chipmunks, eight raccoons, and six Virginia opossum. The percentage (total for a single species/total animals collected) × 100 of wildlife collected on farm 1 was 2.43% for raccoon, 6.39% for Virginia opossum, and 1.82% for eastern chipmunk; on farm 2 it was 1.82% for raccoon, 7.59% for Virginia opossum, and 1.82% for eastern chipmunk; and on farm 3 it was 5.16% for raccoon, 5.77% for Virginia opossum, and 9.72% for eastern chipmunk.

## Discussion

There is a need to better understand tick ecology because of their effects on a variety of animal hosts including humans, wildlife, companion animals, and livestock, but the invasion of *H. longicornis* makes the need more urgent (Dinkel *et al*., 2021). Discerning interactions between invasive and established tick species are imperative for understanding tick community structure on hosts. This study successfully identified wildlife and established tick species associated with the presence of *H. longicornis* which can be targeted for tick surveillance and management. Although ixodid tick species only spend 10% of their total life cycle on hosts compared to the environment (Needham and Teel, 1991), knowing which host body regions are associated with tick attachment allows us to develop important tools for reducing large populations at a single time, which is especially important for a clonal species. In this study, tick and host body regions associated with *H. longicornis* presence that can be targeted for their control were identified. For example, targeting raccoon and Virginia opossum host's pinna, on farms infested with *H. longicornis*, would be an important on-animal control measure for exposing a larger abundance of ticks and tick species to acaracides.

Here wildlife host species types that were associated with tick parasitization but can also be evaluated as targets for on-farm tick control were identified. *Haemaphysalis longicornis* and other tick species were more likely to be present on medium-sized hosts compared to small mammals suggesting that managing these hosts may reduce tick populations on a farm. Raccoons had the highest abundance of *H. longicornis*, the greatest number of cofeeding tick species (6 species total) and the second highest tick Shannon diversity index. The next most heavily parasitized mammal after the raccoon was the Virginia opossum, which had the fourth highest tick Shannon diversity index. In previous studies in Tennessee, >90% of the ticks were collected from these 2 species and raccoons similarly had the highest tick species diversity (Kollars, 1993). The high abundances of *H. longicornis* on these 2 species are concerning because they could serve as reservoir hosts for some *Anaplasma* and *Rickettsia* species (Boostrom *et al*., 2002; Levin *et al*., 2002). Raccoons are known to have large variable home ranges in the Southeast (Hill *et al*., 2023) and females often seek out dens in forested or edge environments near agricultural areas when rearing young (Henner *et al*., 2004). The Virginia opossum is also known to use edge environments and have large home ranges for its medium size (Hill *et al*., 2023). These selection behaviours by raccoon and Virginia opossum coincide with *H. longicornis* habitat preferences (Trout Fryxell *et al*., 2023), suggesting that managing these animals may help prevent the spread and/or establishment of *H. longicornis* on a farm. Differences in the abundances of *H. longicornis* life stages, in which raccoon had more larvae but Virginia opossum had more females, could be due to adult life stages selecting less immature tick-infested hosts (Bloemer *et al*., 1988; Estrada-Peña *et al*., 2020; Tiffin *et al*., 2021).

Eastern chipmunks are known to exist in relatively small home ranges outside of their burrows (Yahner, 1978); therefore, decreased *H. longicornis* tick numbers could be due to fewer eastern chipmunk encounters. Eastern chipmunks also had the highest tick Shannon diversity index; decreased abundances could be due to negative interactions between tick species (Krasnov *et al*., 2007). Although the lowest number of *H. longicornis* was found infesting eastern chipmunks, they can serve as a potential reservoir host for some *Borrelia* and Rickettsiales-related tickborne pathogens (Mclean *et al*., 1993; Keesing *et al*., 2014; Siy *et al*., 2021). Despite collecting *I. scapularis*, *D. variabilis,* and *A. americanum* from rodents, *H. longicornis* were not found on these hosts. *Peromyscus leucopus* had the third highest tick Shannon diversity index, *O. nuttalli* and *P. maniculatus* had the fifth highest, and *P. gossypinus* had the sixth highest. Although *H. longicornis* is known to occasionally feed on rodents, they are not the primary host of this tick species and are not a major concern for transmission of rodent-associated pathogens such as *B. burgdorferi* (Breuner *et al*., 2020; Ronai *et al*., 2020). Additionally, this study found *I. scapularis* to be more abundant on medium-size mammals compared to small mammals.

Overall, raccoons and opossums, had the highest likelihood of having invasive ticks present at the wildlife–livestock interface, which can be isolated for tick management in large abundances, were identified. Baiting regimes focused on these host species could be evaluated for acaracide applications to target *H. longicornis* and other important tick species on Tennessee farm settings since they feed and distribute a number of species. Additionally, raccoons and opossums are also likely to take advantage of livestock operations, by exploiting feed and watering systems, and come into frequent contact with cattle (Atwood *et al*., 2009). Medium-size wildlife could be targeted for on-animal acaracide application by using methods modified from the 4 deer’4-poster’ station (Solberg *et al*., 2003) or systemically with acaracide-treated feed (Pound *et al*., 1996). These methods for controlling ticks on meso mammals should be further studied for tick control. Additionally, culling animals with extremely high population densities could also reduce the number of ticks on sites by decreasing tick host populations (Martin *et al*., 2023). However, for this method, population density counts would need to be well maintained to ensure that a species' populations are well managed.

Different tick species are known to parasitize certain regions of hosts' bodies, including the cervical spine, head, pinna, tail, legs, scrotum, and anus (Koch, 1982; Hart *et al*., 2022). The inclination to parasitize different body regions could be a result of accessing host-seeking/attachment site, attraction to pheromones or host excessive heat, humidity, or protection from grooming (Hair *et al*., 1975; Cardé and Baker, 1984; Shaw *et al*., 2003; Carr and Salgado, 2019; Lydecker *et al*., 2019). In this study, *A. americanum*, *H. longicornis*, *D. variabilis,* or *I. scapularis* interacted together equally on raccoon, Virginia opossum, and eastern chipmunk host's facial regions which also had the second highest average Shannon index of species diversity. Host pinna regions had the highest abundance and greatest tick diversity overall, although *A. americanum* presence outnumbered all other tick species. These results could be due to ticks using negative geotaxis, a mechanism in which ticks walk upwards to find available attachment sites upon encountering a host (Lees, 1948; Kröber *et al*., 2013). Others have noted that tick selection of host pinna regions could be due to inaccessibility of hosts at grooming these areas (Guerin *et al*., 2000). Previous research has identified various volatiles on the host's head, used during mating season, could attract ticks to this body region (Gassett *et al*., 1997). Carr and Salgado (2019) found that ticks use their Haller's organ to detect radiant heat emitted from hosts which may explain the number and diversity of ticks removed from the host's head and pinna regions (Vianna and Carrive, 2005). Some tick species are highly attracted to host body regions that source CO_2_ (Norval *et al*., 1992), which could contribute to our outcome in which a greater abundance and diversity of tick species congregate on the head and pinna regions. Kaur *et al*. (2017) reported that ticks generally chose highly vascular areas on host body regions with thinner skin, ticks infesting hosts head and pinna regions in our study could be due to similar results. Ticks could also be attracted to certain regions of hosts due to females emitting assembly pheromones, which signal clustering of ticks to enhance mating opportunities (Sonenshine *et al*., 1985). Additional research on interactions between native US tick species and *H. longicornis* pheromones should be further investigated.

In our study, the presence of *A. americanum* on a host increased the likelihood that *H. longicornis* was also present. *Haemaphysalis longicornis* followed similar activity and host selection patterns as *A. americanum*, in which all life stages of both species were found predominantly on medium-size mammals but were almost non-existent on small mammals. Other studies investigating *H. longicornis* host use also found similar results in the US (Thompson *et al*., 2021; Tufts *et al*., 2021; White *et al*., 2021). Here, *A. americanum* and *H. longicornis* were found to equally occupy and interact on raccoon and Virginia opossum host's ventrum. Jang *et al*. (2016) also noted the primary attachment sites for *H. longicornis* were the extremities, abdomen, and inguinal region. Cooccurrence of these 2 tick species on these regions may also be related to reduced competition or based on density dependence in available feeding space (Anderson *et al*., 2013). Since these 2 tick species were readily present together on the ventrum and pinna regions had the highest abundance of both tick species, targeting these regions for tick control with an acaracide application could be important for the management of both species. Importantly, these two tick species are also found to have predicted geographic distributions which overlap one another in the US (Raghavan *et al*., 2019; Namgyal *et al*., 2020). Investigations of questing ticks from the environment found a contrasting result where *H. longicornis* larvae and nymphs decreased when *A. americanum* larvae and nymphs increased (Trout Fryxell *et al*., 2023); perhaps suggesting competition at questing sites and not necessarily on the hosts. Future studies are necessary to understand interactions between environmental and host cues associated with tick-feeding patterns.

Contrastingly, the presence of tick species *D. variabilis* decreased the likelihood of *A. americanum* or *H. longicornis* also being present. *Dermacentor variabilis* had a lower presence on the ventrum, pinna, and appendage regions compared to *A. americanum* or *H. longicornis*, but a higher presence on the dorsum compared to *H. longicornis*. *Haemaphysalis longicornis* is found to interact in similar space with *A. americanum* but there appears to be possible competition between *D. variabilis*, which could have serious implications for the spread of tickborne diseases *via* cofeeding (Randolph *et al*., 1996). For example, pathogens associated with *A. americanum* in the US which can be potentially transmitted to *H. longicornis* due to cofeeding include Bourbon and Heartland viruses and *Ehrlichia*, *Anaplasma,* and *Rickettsia* bacteria (Ewing *et al*., 1997; Lee *et al*., 2005; Wright *et al*., 2015; Godsey *et al*., 2016, 2021; Beard *et al*., 2018; Cumbie *et al*., 2022). Future studies which investigate host attributes associated with *H. longicornis* and established tick species on-animal aggregations will be important for understanding tick community structure and pathogen transmission.

*Haemaphysalis longicornis* were more likely to be present on a host as the number of tick species on that host increased; overall, 1–5 tick species cofeeding on hosts were found. Competition between species is often the result of limited shared resources (Mirrahimi *et al*., 2014). In this study, *H. longicornis* presence was predicted to occur when a greater number of tick species were present, suggesting a neutral relationship. In addition, *H. longicornis* was less likely to be present whenever it occurred alone with either tick species *A. americanum*, *I. scapularis,* or *D. variabilis*. Cofeeding ticks can transmit pathogens to one another (Randolph *et al*., 1996). Although a diverse tickborne pathogen community infesting hosts and tick vectors could promote competition between pathogens which ultimately reduces their abundance (Genne *et al*., 2018), tick–pathogen community structure is less studied for *H. longicornis* in the US. Presently, established tick species which can be used to identify the presence of an *H. longicornis* on wildlife hosts were identified. The increased likelihood for *H. longicornis* to be present feeding on a host when multiple tick species are also present at the same time is a concern because the transmission risk is unknown for the cofeeding behaviour.

Importantly, significant IPM strategy effects on the host level; specifically, ticks were less likely to be present on wildlife sampled from farm 1 compared to wildlife sampled from farm 2 or 3, were observed. Previously the management decisions used by producers effectively reduced *H. longicornis* populations from the environment were documented (Butler and Trout Fryxell, 2023), and here, those management effects were seen on wildlife as well, fewer *H. longicornis* ticks were on wildlife from farm 1. The percentages for raccoon, opossum, and eastern chipmunk caught at each farm were also similar although the total number of wildlife species varied. Understanding interactions between the environment, hosts, tick population dynamics, and human exposure is important for creating a tick IPM strategy (Stafford *et al*., 2017). Mechanical, cultural, and on-animal chemical control can reduce *H. longicornis* in the environment and on wildlife in a farm setting.

Different mammal host and tick species characteristics which can be targeted for control of an invasive tick at the wildlife–livestock interface were identified. Future studies may investigate on-host management regimes in the field to reduce *H. longicornis*. These could include culling or acaracide-baiting regimes to target medium-size hosts (raccoons and opossum), or behaviours associated with these hosts during periods such as mating season using attractants; however, these methods need to be evaluated. These medium-size hosts could also be targeted by directing management towards farm practices that allow access to livestock food. The likelihood that *H. longicornis* was present on a host was associated with the presence of multiple species feeding concurrently. Tick control that can target a greater number of tick species may help simultaneously reduce both invasive and established tick species. Identifying activity periods for when established tick species are feeding simultaneously could be important for future *H. longicornis* management. Subsequent research could focus on how semiochemicals on hosts affect tick-feeding strategies. This study identified host and tick variables which were associated with *H. longicornis* at the wildlife–livestock interface. In addition, previous research including producer led IPM strategies in the environment was identified that reduced the likelihood that ticks would be present on wildlife hosts. Although evaluation before culling animals is recommended, future studies should investigate *H. longicornis* management by targeting these variables to reduce tick presence.

## Data Availability

Data will be made available upon request from corresponding author.
